# A qualitative investigation of facilitators and barriers to DREAMS uptake among adolescents with grandparent caregivers in rural KwaZulu-Natal, South Africa

**DOI:** 10.1371/journal.pgph.0000369

**Published:** 2022-09-22

**Authors:** Dumile Gumede, Anna Meyer-Weitz, Thembelihle Zuma, Maryam Shahmanesh, Janet Seeley

**Affiliations:** 1 Centre for General Education, Durban University of Technology, Durban, South Africa; 2 Africa Health Research Institute, KwaZulu-Natal, Durban, South Africa; 3 School of Applied Human Sciences, University of KwaZulu-Natal, Durban, South Africa; 4 Institute for Global Health, University College London, London, United Kingdom; 5 London School of Hygiene and Tropical Medicine, London, United Kingdom; Universitas Sebelas Maret Fakultas Kedokteran, INDONESIA

## Abstract

Adolescents with grandparent caregivers have experienced challenges including the death of one or both parents due to HIV in sub-Saharan Africa. They may be left out of existing HIV prevention interventions targeting parents and children. We investigated the facilitators and barriers to DREAMS (Determined, Resilient, Empowered, AIDS-free, Mentored and Safe) programme uptake among adolescents with grandparent caregivers across different levels of the socio-ecological model in rural South Africa. Data were collected in three phases (October 2017 to September 2018). Adolescents (13–19 years old) and their grandparent caregivers (≥50 years old) (n = 12) contributed to repeat in-depth interviews to share their perceptions and experiences regarding adolescents’ participation in DREAMS. Data were triangulated using key informant interviews with DREAMS intervention facilitators (n = 2) to give insights into their experiences of delivering DREAMS interventions. Written informed consent or child assent was obtained from all individuals before participation. All data were collected in isiZulu and audio-recorded, transcribed verbatim and translated into English. Thematic and dyadic analysis approaches were conducted guided by the socio-ecological model. Participation in DREAMS was most effective when DREAMS messaging reinforced existing norms around sex and sexuality and when the interventions improved care relationships between the adolescents and their older caregivers. DREAMS was less acceptable when it deviated from the norms, raised SRH information that conflicts with abstinence and virginity, and when youth empowerment was perceived as a potential threat to intergenerational power dynamics. While DREAMS was able to engage these complex families, there were failures, about factors uniquely critical to these families, such as in engaging children and carers with disabilities and failure to include adolescent boys in some interventions. There is a need to adapt HIV prevention interventions to tackle care relationships specific to adolescent-grandparent caregiver communication.

## Introduction

The HIV epidemic and the high mortality of the biological parent generation have left many adolescents in the care of grandparents in sub-Saharan Africa (SSA) [1]. In 2019, the General Household Survey in South Africa found that 3.1% of children (0–17 years old) were maternal orphans, 9% of children were paternal orphans, and 2.4% of children were double orphans [2]. The percentage of orphaned children in KwaZulu-Natal was 18.7% and one of the highest in the country [2]. Adolescents (10–19 years old) with grandparent caregivers are a vulnerable and critical population in efforts to prevent HIV acquisition.

The death of one or both parents due to HIV can impact the adolescents’ well-being throughout their lifetime [3] and increase their vulnerability to HIV and risky behaviours [4]. It also has a significant influence on their care arrangements due to household dissolution and migration [5]. The burden of care for these adolescents has often been undertaken by grandparents [6]. Grandparent caregiving can occur suddenly or after a prolonged illness of biological parents [7]. While HIV care and antiretroviral therapy (ART) services have significantly reduced AIDS-related deaths [8], large numbers of orphaned and vulnerable adolescents are still cared for by grandparents in South Africa and elsewhere in SSA [6, 9].

Being raised by grandparent caregivers can be difficult for adolescents, considering that many developmental, psychological, social, and structural transitions converge in this period of their life [10]. It is a development phase characterized by increased risk-taking and greater peer influence in decision-making [11]. During this development phase, new boundaries are explored, and caregiver rules and societal norms that were unchallenged during childhood are re-examined, questioned, and may be challenged in preparation for adulthood [12]. Adolescents with grandparent caregivers have particular challenges which other adolescents may not face. These adolescents face more socio-economic risks including poverty that can affect their development in very unique ways than other adolescents [13]. Researchers have reported that these adolescents are usually at increased risk of poorer health and educational outcomes compared to other adolescents [14]. These challenges place adolescents at increased risk for emotional and behavioural problems [7] as well as loss of social opportunities [6, 15].

Given that young people aged 15 to 24 years represent the largest population living with HIV in South Africa, several studies have explored sex communication between adolescents and their caregivers [16–18]. These studies are based on the premise that effective adolescent-caregiver communication on sex is a protective factor in adolescent risky sexual behaviours [17, 19]. For example, a study conducted in KwaZulu-Natal of adolescents and their caregivers participating in a family-centered HIV prevention intervention found that adolescents’ HIV and condom use knowledge significantly improved [17]. However, South African studies also state that adolescent-parent communication about sex is often negative or punitive [20, 21], which might therefore hinder open communication about minimizing unsafe sexual behaviour [22], with the generation gap between older and younger generations blocking sexuality communication [23]. This gap may be further exacerbated when the carers are grandparents. While many of the previous studies have focused on adolescent-parent communication, adolescent-grandparent communication especially around sexuality and HIV can be difficult yet not much is known about their circumstances. Thus, the adolescents with grandparent caregivers are particularly vulnerable to acquiring HIV [24] and very little is known about how they communicate about sexuality and HIV with their grandparent caregivers.

While HIV prevention interventions are increasingly targeting adolescents in general [25], very few interventions are sensitive to the many adolescents with grandparent caregivers in South Africa. This group of adolescents are a uniquely challenged generation and their invisibility in policy framing could imply that they are left out of existing HIV prevention interventions. Understanding the dynamics and contextual idiosyncrasies shaping the experiences of adolescents with grandparent caregivers is important for identifying factors that impact decision-making relating to initiating sexual activity and preventing pregnancy. Being aware of the issues that influence adolescents’ lives in grandparent families and their participation in HIV prevention interventions can help implementers provide improved, family-oriented and result-driven services.

In recent years, there has been increased public health interest in offering HIV prevention interventions for adolescents [26–29]. However, little is known about the contextual factors that shape the participation of adolescents living with grandparent caregivers in HIV prevention interventions. The socio-ecological model (SEM) acknowledges interrelated individual and contextual factors that influence behaviours [30]. It is described by using multilevel circles that show the individual in the centre and surrounded by the family, community, organisational, and public policy [30]. The behavioural influence of the interactions across the different levels has been discussed by a great number of authors in literature [26, 31, 32]. For example, a recent study in South Africa suggests that socio-ecological factors influenced young people’s intention to access and utilize health services [26]. Although there are many studies, the research on the socio-ecological factors that shape the participation of adolescents with older caregivers in HIV prevention interventions remains limited. To fill this literature gap, in this paper, we investigated the facilitators and barriers to participation in HIV prevention interventions among adolescents with grandparent caregivers across different levels of the SEM covering individual, interpersonal, community and organisational components in the context of DREAMS programme in rural South Africa.

## Methodology

### Study setting

This study was conducted in uMkhanyakude district of northern KwaZulu-Natal, South Africa. The district is predominantly rural with high rates of poverty and unemployment [33]. In this setting, the HIV prevalence and incidence are high [34]. About 19% of adolescent girls and young women (AGYW) and 5.6% of adolescent boys and young men (ABYM) are living with HIV [35].

Within this context, a multi-sectoral HIV combination prevention programme–the DREAMS programme was implemented between April 2016 − September 2018 to reduce HIV infection in AGYW (and their male sexual partners) through evidence-based health, educational and social interventions [34]. The DREAMS programme is an investment by the U.S. Government President’s Emergency Plan for AIDS Relief (PEPFAR) office, Bill and Melinda Gates Foundation, Girl Effect (formerly the Nike Foundation), and other private sector partners, announced in 2014 [36]. The overall aim of DREAMS was to reduce HIV incidence in AGYW through a combination of interventions that target community, family, male partners, and AGYW to promote safer sexual relations, social protection, biological protection, and empower AGYW in 10 countries in sub-Saharan Africa including South Africa [11]. The DREAMS programme had many components, as described elsewhere [37], that were delivered through different DREAMS implementing partners. The DREAMS implementing partners, sometimes, delivered DREAMS through local community-based organisations (CBOs). UMkhanyakude district is one of the sites in which the DREAMS programme was implemented in South Africa [34].

### Study design, sampling and study participants

Adopting a qualitative method study design, we selected a purposive sample of adolescents aged 13–19 years, grandparent caregivers aged 50 years and over, and DREAMS intervention facilitators to painstakingly collate data for this study. Key inclusion criteria were (a) adolescents (boy or girl) aged 13–19 years who were the recipient of at least one DREAMS intervention and their primary caregivers were grandparents (male or female) aged 50 years and above, and (b) DREAMS intervention facilitators (male or female) working for a community-based organisation that was subcontracted by a DREAMS implementing partner for the delivery of DREAMS interventions in the study area.

### Data collection

Repeat in-depth interviews were conducted in the rural community of Mtubatuba sub-district with adolescent-grandparent caregiver dyads and key informant interviews with DREAMS intervention facilitators. Interview guides were used, which consisted of a series of open-ended semi-structured questions tailored for each category of the study participants, to gain insight into the DREAMS interventions and factors shaping adolescents’ participation in DREAMS. Separate interviews were conducted with adolescents and their caregivers to allow each dyad partner to express beliefs and perceptions more freely. Data collection was spread through three phases, at every four months, over 12 months from October 2017 to September 2018 in order to elicit rich qualitative data and for prolonged engagement. Each one-on-one interview was carried out in an undisturbed and private setting of choice, for example, in the participants’ homes or their gardens. The first author (DG) is a social scientist trained in qualitative data collection and speaks both isiZulu (the local language of the participants) and English fluently. DG conducted all the interviews, audio-recorded, transcribed verbatim and translated all data into English. TZ, a local senior social scientist, checked for the accuracy of translations and cultural appropriateness. Throughout the interviews, field notes were kept to detail pre- and post-interview reflective thoughts, observations and impressions.

### Data analysis

Translated data were coded and managed using Atlas.ti 8. Both thematic [38] and dyadic [39] approaches were used in the analysis. The first author (DG) conducted the main analysis of the interviews to develop the initial coding framework based on the levels of the socio-ecological model and salient issues that arose in the data. Over a series of analytical meetings with the researchers (AMW, TZ, MS and JS), the final coding framework was developed. The debriefing also facilitated reflexivity and exploration of alternative explanations. When coding was complete, DG developed data matrices which highlighted prominent themes relevant to adolescents’ facilitators and barriers to DREAMS. Data from the field notes were also used to further inform the development of themes. To increase the rigour of the analysis, data source triangulation, prolonged engagement and debriefing [40] were adopted in this study. Quotes were extracted from the transcripts to illustrate common responses. COREQ guidelines for qualitative papers were followed.

### Ethical considerations

The University of KwaZulu-Natal Humanities and Social Sciences Research Ethics Committee (ref. HSS/1109/017D) reviewed and approved the study, along with approval from the Community Advisory Board (CAB). Individuals provided written informed consent before they participated in the study; however, assent and caregiver written consent was obtained for all adolescents aged below 18. No names of participants were recorded; instead, pseudonyms were used.

## Results

### Characteristics of study participants

A total of 36 repeat in-depth interviews were conducted with adolescent-grandparent caregiver dyads (n = 12). Participants’ characteristics are summarized in Table 1. Of the six adolescents, five were adolescent girls and one was an adolescent boy aged between 13 and 19 years. Each adolescent received one or more DREAMS participatory group training interventions through a local CBO. These interventions were Stepping Stones, Let’s Talk, and Vhutshilo.

**Table 1:** Characteristics of adolescent-grandparent caregiver dyads.

Adolescents (n = 6)						Grandparent caregivers (n = 6)		
Name^a^ and age (years) in 2017	Sex	DREAMS Intervention	In school	Biological mother alive	Biological father alive	Name and age (years) in 2017	Sex	Relationship
Thabani (15)	M^b^	Stepping Stones	Y^d^	Y	Y	MaNdawo (76)	F	Paternal grandmother
Neli (14)	F^c^	Let’s Talk	Y	Y	N	MaZulu (64)	F	Maternal grandmother
Zama (15)	F	Let’s Talk	N^e^	Y	Y	MaNgubo (80)	F	Maternal grandmother
Sane (13)	F	Stepping Stones, Let’s Talk	Y	Y	N	MaDube (58)	F	Maternal grandmother
Thandi (13)	F	Let’s Talk, Vutshilo	Y	Y	N	MaJali (56)	F	Maternal grandmother
Mpume (19)	F	Let’s Talk	N	Y	N	MaKhoza (64)	F	Paternal grandmother

^a^All names are pseudonyms.

^b^M = Male.

^c^F = Female.

^d^Y = Yes.

^e^N = No.

The Stepping Stones intervention focuses on improving gender equity and communication and relationship skills [41]. The Let’s Talk is a structured, manualized, small-group HIV prevention intervention with separate and joint sessions for adolescents age 13 or older and their caregivers and is designed to assist in effective communication between caregivers and adolescents, strengthening family relationships, and mitigating adolescent sexual risk [42]. In the same way, the Vhutshilo is a curriculum designed for orphaned and vulnerable adolescents, delivered by peer educators from the same background and community, to provide HIV prevention skills and psychosocial support [43].

With regard to their grandparent caregivers, all of them were older women aged 56 to 80 years. Four were maternal grandmothers and two paternal grandmothers to the focal adolescents.

Additionally, one male aged 30 (Stepping Stones intervention facilitator) and one female aged 41 (Let’s Talk intervention facilitator) were key informants, and they were both employed by the local CBO to deliver DREAMS interventions in uMkhanyakude district.

### Facilitators and barriers to DREAMS participation using the socio-ecological framework

**Fig 1:**
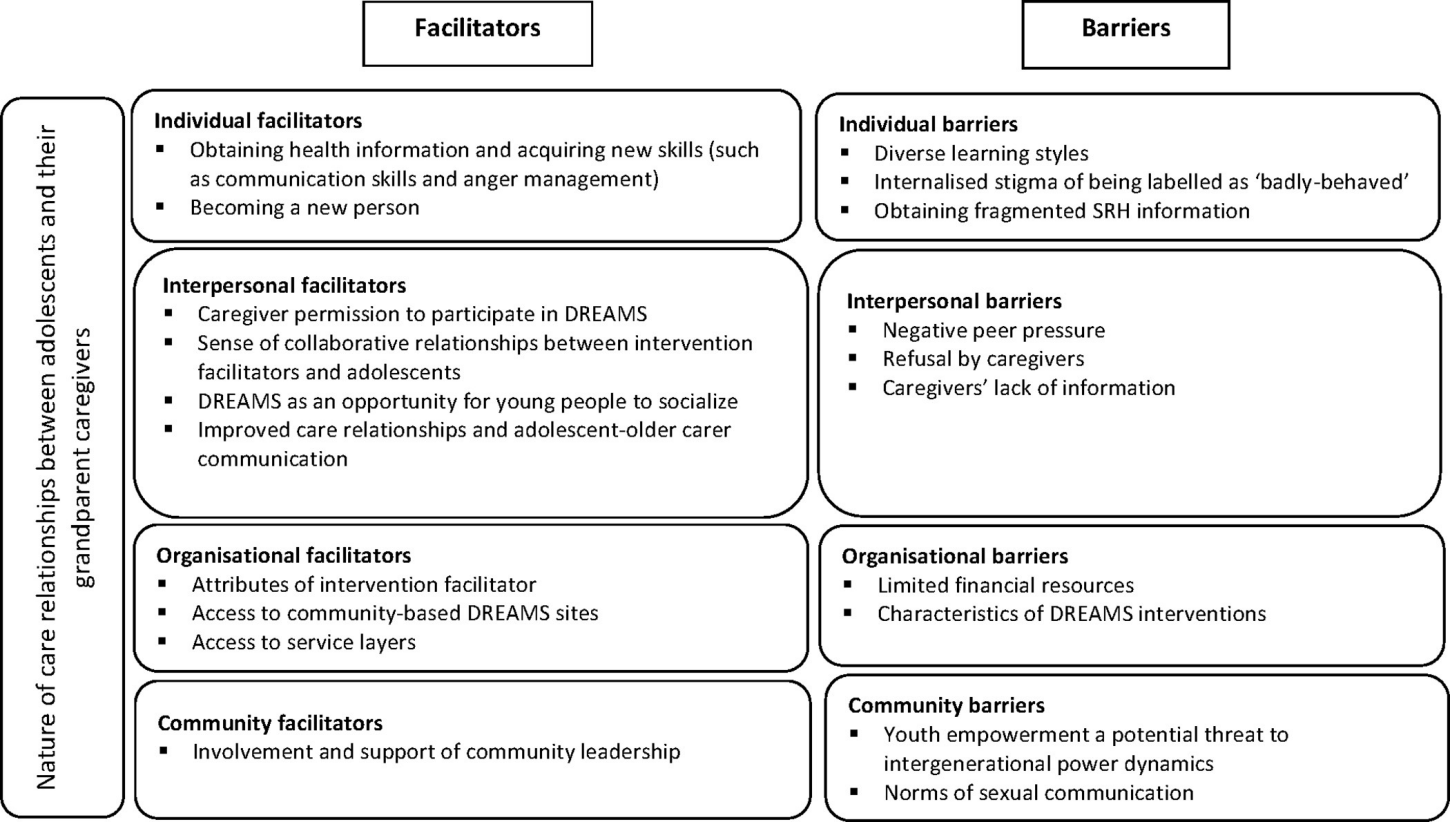
Facilitators and barriers to DREAMS participation among adolescents with grandparent caregivers.

The facilitators and the barriers to participation in DREAMS HIV prevention interventions among adolescents in older carer families are organised into four levels of the socio-ecological model (SEM) namely individual, interpersonal, organisational, and community levels, as indicated in Fig 1. For each level, themes are supported by illustrative quotes from the interviews.

### Individual-level factors facilitating adolescents’ participation in DREAMS interventions

#### Obtaining health information and acquiring new skills

Some adolescents mentioned that obtaining relevant information regarding HIV and health issues motivated them to participate in DREAMS as these were contextual issues affecting young people. They listed topics which they discussed with intervention facilitators during DREAMS sessions. Broadly, these topics included HIV, sexually transmitted infections (STIs), Tuberculosis (TB), condoms, contraceptives, communication, and career planning.

*We learnt a lot*. *We learnt about many STIs*, *the names of STIs*, *how STIs are contracted*, *and the link between STIs and HIV … I discovered that there are many diseases besides TB and HIV*. *… We learnt about families with financial problems*, *relationships with other people*, *and people to contact for help*. *The programme taught us real life issues faced by young people such as family relationships and handling family conflicts*. *Most of the time*, *the topics were very important to us*. *They touched on things that I have seen and experienced*. *Like*, *I’ve asked myself what do I want for my future*? *What steps do I need to follow*? *… I believed that I will learn something new in each session to change my life for the better hence I encouraged myself to continue participating in the programme*. *(Thabani*, *boy*, *15yrs*)

It appears from Thabani’s narrative that the factors motivating participation in DREAMS went beyond acquiring relevant health-related information to include gaining knowledge participants described as relevant for ensuring harmonious family relations and stability. In the same way, the DREAMS intervention facilitators also affirmed that the adolescents enjoyed discussing topics such as condoms, sexual relationships, and career planning during the DREAMS participatory sessions.

In addition, the DREAMS intervention facilitators mentioned that the topic of anger management revealed that the adolescents were dealing with anger issues regarding gender inequalities at home and resented their parents’ sexual relationships with new partners other than their biological parents.

*…topics on condom use and sexual relationships*. *They* [adolescents] *liked those topics*! *And the session about gossiping and proper communication*. *The anger management session raised emotions amongst the adolescents*…*Oh*, *they have anger issues*! *You find a parent thinking that everything is alright with her girl child while the girl child is angry with the parent because the girl child feels the parent loves the boy child more than the girl child*. *There are things that parents do to the girls but not to the boys*. *Sometimes boys do wrong things intentionally and are fully aware that they won’t be punished like girls*. *Anger and frustration start building up inside a girl’s mind*. *Another source of anger is a mother who has a [*sexual*] relationship with another man* [stepfather] *… the child becomes jealous*, *angry and full of hatred*. *(Let’s Talk intervention facilitator*, *female*, *41yrs*)

Further, the DREAMS intervention sessions provided the opportunity for the adolescents to talk about negative caring behaviours of their caregivers such as unequal gender treatment at home. The opportunity to talk about gender inequalities at home raised emotions for the adolescents. However, learning about anger management in DREAMS interventions equipped the adolescents with skills to control negative emotions in communication, and in adjusting to parents’ sexual relationships, considering that the biological parents of the adolescents that participated in the study were no longer in the relationship.

#### Becoming a new person

Some adolescents mentioned that they reflected on themselves and saw a changed version of who they are in terms of their abilities in choosing friends and setting boundaries. One adolescent explained that participating in DREAMS assisted her to redefine the types of friends she wanted and to setting the limits with her friends in terms of what was acceptable and unacceptable towards her:

*I used to have bad friends who enjoyed wandering in the streets*, *not doing household chores and being disrespectful towards adults*. *My grandmother doesn’t like me to associate with bad friends*. *In group sessions* [DREAMS], *we were taught how to choose good friends for us*. *Initially*, *it was difficult to dump my old friends but I realised they were not good for me and no longer fitted my new criteria of friends*. *While I was bored and lonely without my old friends*, *but*, *gradually*, *I learnt new skills to choose good friends*. *Now*, *I surround myself with friends who do household chores at their homes and who show respect to adults … it’s very important to learn to be surrounded by good friends otherwise I would have changed to be as bad as my old friends…even my grandmother would have been very angry with me for hanging out with bad friends*. *(Thandi*, *girl*, *13yrs*)

It seems that DREAMS interventions also reinforced some behaviours and values such as showing respect to adults that the grandparents found acceptable. There was a sense that where the messages taught by DREAMS initiatives align with normative cultural ideas upheld by grandparent caregivers, then what was considered positive change was more likely to happen.

Others mentioned they believed they had changed from behaviours that were considered risky and those that were regarded as unacceptable due to participation in DREAMS. They constructed reflections of themselves pre-and during DREAMS participation. From their reflections, they believed that pre-DREAMS participation they had the tendency of teaming up with bad friends to engage in risky behaviours such as flirting to get money from older men and unacceptable behaviours such as not doing household chores. It seems that hanging out with bad friends was associated with leading to engagement in risky and unacceptable behaviours. However, during DREAMS participation, they believed they overcame the tendency of being negatively influenced by bad friends to engage in risky behaviours. They were, thus, motivated to participate in DREAMS due to seeing themselves becoming a new version of themselves.

However, the dyadic analysis revealed some similarities and differences in perspectives between some adolescents and their grandparent caregivers regarding whether the adolescents had adopted acceptable behaviours or not. For example, one adolescent stated that she used to flirt to get money from older men at the taverns and slept away from home, although she did not admit to having sex with the men she seduced for money. However, she went on to mention that she transformed into a new person through her participation in DREAMS:

*Initially*, *there was no difference in the way I behaved*. *But during the course of DREAMS*, *I changed because they taught us how to behave like young people*. *Here at home*, *I changed into a new person*, *I became a well-behaved person*! *I used to go out a lot and returned at night*. *Sometimes I didn’t come back home*. *I went with my friends to the taverns to rip off money from men* [patrons]. . . . *My friends were hustlers*. *They smoked dagga and drank alcohol*. *… Oh*, *I no longer do that now*. *My friends were even complaining and saying to me*, *‘you are boring us for not going with us to the tavern anymore’*. *I think the reason*, *I no longer go to the tavern*, *is the lessons I learnt from DREAMS*…*DREAMS changed me into a well-behaved person*. *(Zama*, *girl*, *15yrs*)

Similarly, in a separate interview with her grandmother, she confirmed that there was a big difference in her granddaughter’s behaviour at the time she participated in DREAMS. She abstained from being absent a lot from home and spent more time at home with the family. The grandmother was happy about the changes she saw in her granddaughter, and this improved their relationship:

*I can say those classes were helpful*. *She stopped wandering out and stayed with me here at home*. *She became useful with household chores*. *I was also at peace to see her at home rather than not knowing where she was at night*. *I could sleep peacefully at night knowing that she is safe*. *(MaNgubo*, *older carer*, *80yrs*)

It was clear that both the adolescent and her grandparent caregiver agreed that the adolescent had changed her behaviour of sneaking out of the house and sleeping away from home. Pre-DREAMS, they had a difficult relationship due to the adolescent’s unacceptable and risky behaviours. Moreover, the transformation that occurred in the adolescent’s behaviours improved the care relationship as they spent time bonding at home.

While some dyads agreed that participation in DREAMS had transformed the adolescents to adopt acceptable behaviours, the other dyads did not have a similar position on this. An example of a disagreement is seen between Thabani and his grandmother. According to Thabani, he perceived himself as having developed the ability to control his negative emotions and attitudes:

*We did a session about financial problems within families*. *I used to be very angry when my father and I could not communicate about my needs and when he refused to provide me with the things I needed*. *I would be upset and angry to such an extent that I took my anger out on my parents*. *But DREAMS taught me that I needed to be humble and accept the things that I cannot change*. *For example*, *it didn’t mean that I was not going to pass my exams at school for not getting money for a school trip*. *(Thabani*, *boy*, *15yrs*)

However, in a separate interview, his grandmother had a contrary view of her grandson and said that she did not notice a difference in his ability to manage anger:

*Nothing has changed with Thabani*. *He is still the same as he was*. *Of course*, *he doesn’t go out in the night*, *but*, *during the day*, *he still goes out where he likes*, *as he likes*. *He’s just defiant*. *It’s difficult to reprimand him*. (*MaNdawo*, *older carer*, *76yrs)*

It appeared that the members of the dyad had different perceptions of how DREAMS shaped the adolescent’s behaviours. The boy thought that he had become a better person as a result of participating in the intervention while his grandmother felt he had not changed the behaviours that were considered unacceptable.

### Interpersonal level factors facilitating adolescents’ participation in DREAMS interventions

#### Caregiver permission to participate in DREAMS

Caregiver permission was one of the factors that facilitated the participation of adolescents with older caregivers in DREAMS.

*I went to attend the classes* [DREAMS] *because she* [Let’s Talk facilitator] *requested permission from my grandmother to join the programme and my grandmother granted the permission*. *(Thandi*, *girl*, *13yrs)*

[Let’s Talk facilitator] *visited my grandmother here at home to tell her about the classes* [DREAMS], *then I joined the programme upon my grandmother’s permission*. *(Mpume*, *girl*, *19yrs*)

Culturally, adolescents could not consent to participate in HIV prevention interventions and thus the caregiver permission necessitated their participation in DREAMS.

#### Collaborative relationships between intervention facilitators and adolescents

A sense of collaborative relationships that developed between the adolescent DREAMS recipients and the intervention facilitators over the course of DREAMS positively contributed to the retention of adolescents in the interventions. One intervention facilitator shared his experience that being able to build rapport with the adolescents made them more willing to attend and actively participate in DREAMS:

*We talked after every session and motivated the young people to continue participating in the programme*. *We engaged with them until we became friends*. *Building friendships with these young people was our strategy to motivate them to remain in the programme*. *They were open with us*. *Even when we meet in the community*, *they asked ‘when are we meeting for a learning session*?*’ Truth be told*, *we discussed things that are not included in the school curriculum*. *And the sessions were facilitated by young adults*. *So*, *they*, *basically*, *viewed me as one of them*. *(Stepping Stones intervention facilitator*, *male*, *30yrs*)

It was evident that the intervention facilitators endeavoured to nurture good relationships with the adolescents in promoting their uptake in DREAMS interventions and emphasizing safe social spaces for the adolescents to tackle health and social issues affecting their lives.

#### DREAMS as an opportunity for young people to socialize

In addition, adolescent participation in DREAMS provided an opportunity for the adolescents to socialize with other young people as they connected with peers and friends in a safe space. Many adolescents came along with their friends to participate in DREAMS and, thus, created a sense of cohesion and belonging among the adolescents when they were amongst their friends:

*I liked it because my friends were there*. *We talked and ate biscuits together*. *(Sane*, *girl aged 13yrs*)

*It was nice to meet with my friends during the classes* [DREAMS]. *I really enjoyed attending DREAMS sessions with my friends*. *We played and had fun*. *(Neli*, *girl*, *15yrs*)

*I loved coming* [to DREAMS sessions] *because my friends were there*. *We would really talk and laugh*. *(Mpume*, *girl*, *19yrs*)

Thus, participation in DREAMS created a friendly space for the adolescents to enjoy themselves away from home. It is important to note that the adolescents in older carer families mentioned that the older carers restricted the adolescents’ movements and choice of friends. It was clear that these adolescents had limited opportunities to socialize with their peers as they had responsibilities at home helping older carers and, sometimes, caregivers of their older carers as they had chronic illnesses. As a result, participating in DREAMS brought an opportunity to get away from the home environment and be with friends to have fun, to play games, and to enjoy refreshments.

The DREAMS intervention facilitators also shared similar sentiments that adolescents were motivated to participate in the interventions in order to meet with friends:

*They enjoyed being together as peers to play and learn about different topics*. *They were fascinated by games*, *role plays and condom demonstrations; hence they didn’t want to be absent for sessions*. *(Stepping Stones intervention facilitator*, *male*, *30yrs*)

It seemed DREAMS provided the opportunity for the adolescents to disengage from school work, and housework and they were free from caregiver control. This implies that adolescents desired a space to socialize with other young people.

#### Improved care relationships and adolescent-older carer communication

One of the main factors facilitating participation in DREAMS was that it improved care relationships and communication between the adolescents and their grandparent caregivers. One of the DREAMS interventions, Let’s Talk, seems to be playing an important role in strengthening communication between some adolescents and their older carers. This was illustrated by one adolescent who participated in the Let’s Talk intervention together with her grandmother:

*There was a difference in our communication*. *She used to shout at me and not listen to what I needed to say*. *I can say my grandmother was able to listen to me until I finished when talking to her … sometimes older people don’t want to listen to the young ones and it’s difficult to talk when they don’t want to listen*. *It’s like you don’t respect them and you are badly-behaved … although*, *we still talk but I feel we talked a lot during DREAMS*. *(Mpume*, *girl*, *19yrs*)

It seemed the generational gap between the adolescents and their grandparent caregivers shaped the nature of communication between them. The adolescents mentioned fear of expressing their feelings to their grandparent caregivers in order to avoid being labelled as ‘badly-behaved’ or disrespectful to adults. Participating in DREAMS appeared to enable the dyads to discuss issues openly and share their feelings on different aspects.

According to the Let’s Talk intervention facilitator, the intervention offered separate sessions for the adolescents and for the caregivers/parents as well as joint sessions for both the pairs. Only two adolescents in this study participated in the Let’s Talk intervention with their older carers.

*During the joint session*, *caregivers shared their experiences about behaviours they don’t like from their children*, *and young people did the same about what they don’t like from their caregivers*. *During one of the sessions*, *one adolescent said*, *‘I don’t want my mother to open my bag and judge me about what she found inside my bag*. *I would appreciate it if my mother could ask me when she found something she did not understand in my bag*, *rather than shout at me’*. *This caregiver asked for an apology from her daughter*. *We were so excited that the caregivers were learning how to communicate properly with their children*. *(Let’s Talk intervention facilitator*, *female*, *41yrs*)

Further, the adolescents shared that participation in DREAMS resulted in them being more obedient and respectful towards adult authority. Across the dyads, the older carers emphasised that they expected respect from their adolescent grandchildren, which the adolescents understood as implying ‘*obeying adults and doing as instructed*’. One adolescent illustrated that her participation in DREAMS was motivated by respect for her grandmother in order to avoid conflict in the relationship with her:

*One day I was just lazy to attend the group session but the thought of facing discipline made me to go*. *I went because I knew my aunt and grandmother would check with* [Let’s Talk intervention facilitator] *if I attended the session or not*. *If I had not gone*, *[*Let’s Talk intervention facilitator] *would have told my grandmother and she would have shouted at me for disrespecting her*. *I hate being yelled at*. *(Zama*, *girl*, *15yrs*)

It was clear that Zama continued participating in DREAMS to avoid conflict between her and her grandmother and prioritized maintaining a positive relationship with her grandmother.

One dyad shared how DREAMS improved their communication and the care provided by the older carer to the adolescent. The older carer said that participating in the Let’s Talk intervention taught her to see things from the adolescent’s perspective, leading to improved communication and a better relationship:

*They taught us about the rules of parenting*, *which say we must not act like lions towards our grandchildren*. *We must not shout at our grandchildren when they go out with their friends*. *We must not lockdown our grandchildren at home*. *… Do I mean my granddaughter* [Sane] *must not relate with other children*? *Who will she relate with*? *She is young and I am old*. *I can’t play with her*. *… We learnt those lessons*. *Be polite to a child even when he/she has done a mistake*. *Sit down with the child and talk politely*, *not yelling at the child*. *In future*, *the child might fail to talk to you as a caregiver when he/she encounters a problem*. *The child would be scared of being shouted at … I realised that it is a mistake to act like a lion towards my grandchildren*. *You may think you are old yet there are things which you don’t know*. *Old ways are an enemy to a child*, *nowadays … I had talked to my granddaughter after we had been taught there* [DREAMS]. *I said*: *‘HIV is found through sex*. *Pregnancy is found through sex*. *There is no need for an unplanned child that will end up being a burden to me’*. *She said*: *‘I will never fall pregnant’*… *I last talked with her after we had finished attending the programme*. *Now*, *[*since DREAMS ended] *it’s difficult to talk to her because she doesn’t want to listen*. *As I’ve told you she’s sleeping away from home and misbehaving*. *(MaDube*, *older carer*, *58yrs*)

Likewise, in a separate interview with Sane, she concurred with her grandmother:

*We used to talk a lot with my grandmother as we were taught there* [Let’s Talk]. *We talked about HIV and how it is passed from one person to another*. *Attending the programme helped us to know information that we didn’t know such as how diseases spread*. *Engaging in open conversations with my grandmother also helped me not to do it* [sex]… *unfortunately*, *we don’t talk a lot anymore*. *We were only talking a lot while we were still attending DREAMS*. *(Sane*, *girl*, *13yrs*)

As a result of participation in the Let’s Talk intervention, it seems that engaging in open conversations about sex and HIV between the adolescents and their older carers was enhanced and resulted in more positive caring relationships. Unfortunately, a platform for effective communication between the adolescents and their older carers was not sustained since DREAMS ended.

### Organisational level factors facilitating adolescents’ participation in DREAMS interventions

#### Attributes of DREAMS intervention facilitator

Some older caregivers described the positive qualities exhibited by the DREAMS intervention facilitator that enabled the grandparents to support the participation of their adolescent grandchildren in DREAMS. She was described as a respected community member, a local pastor’s wife, and a co-founding member of a community crèche. Many older carers regarded her as trustworthy, a reliable source of information, having a special ability to work with community members, and being responsive to their needs. This trust was displayed in interviews with the older carers:

[Let’s Talk facilitator] *will never mislead me*! *I trusted that she would not tell us lies and that DREAMS would help my granddaughter to be a well-behaved child*. *She lives with us in the community*. *We respect her as a pastor’s wife … that’s why I had no problem for Sane to attend the classes*. *(MaDube*, *older carer*, *58yrs*)

*We knew* [Let’s Talk facilitator] *a long time ago*. *She looks after young children at the crèche*. *Sometimes*, *when we have family problems*, *she comes in to help us resolve our issues*. *… She told me about the classes* [DREAMS] *and asked for my granddaughter to join*. *I*, *then*, *agreed because she is active in community development projects*. *(MaJali*, *older carer*, *56yrs*)

The perceptions that the grandmothers had about the intervention facilitator played a significant role in the grandmothers to associate DREAMS as a solution to adolescent risk behaviours and community development.

#### Access to community-based DREAMS intervention sites

Community-based DREAMS intervention sites were convenient and easily accessible, thus facilitating the participation of adolescents in the interventions.

*We delivered sessions in local venues in the community*. *The venues were closer to the adolescents’ homes… We also grouped young people based on their place of residence so that they didn’t have to travel long distances from their homes to the implementation sites*. *(Stepping Stones intervention facilitator*, *male*, *30yrs*)

*We attended classes at* [name of venue]. *It took me just a few minutes to get there from home*. *I was basically never late because it’s very close to my home*. *(Neli*, *girl*, *14yrs*)

Older carers were also comfortable knowing that their adolescent grandchildren were in the neighbourhood as they were concerned with the safety of the adolescents, as one stated:

*I’m happy that the classes* [DREAMS sessions] *were delivered closer to my house*. *It was safer for Thandi to get there*. *(MaJali*, *older carer*, *56yrs*)

#### Access to service layers

Access to HIV testing and other services which were offered by the CBO implementing DREAMS interventions also facilitated the uptake of DREAMS by the adolescents. One DREAMS intervention facilitator explained that sometimes some adolescents were transferred from one intervention to another within the organisation if the adolescents appeared not ready to exit DREAMS. The local CBO also collaborated with other service organisations to refer the DREAMS recipients for additional services. Some of the services mentioned were HIV Testing Services (HTS), TB screening and testing, and contraceptives.

*We were referred* to [name of organisation] *for HIV testing*. *… I did go and was tested for HIV*. *(Sane*, *girl*, *13yrs*)

*I’ve always wanted to know my HIV status*. *We were even told that we could go to* [name of organisation] *if we want to test for HIV*. *So*, *I went with other girls to test for HIV*. *(Neli*, *girl*, *14yrs*)

The local CBO that delivered DREAMS interventions also provided wider social and health services in the community. While delivering DREAMS interventions, the organisation also linked the adolescents with government departments to obtain birth certificates and child support grants. One adolescent stated:

*They helped me to get the child support grant since my grandmother had struggled for a long time to apply for the grant as my mother’s whereabouts are not known and she is nowhere to be found*. *(Mpume*, *girl*, *19yrs*)

Access to HIV testing and the additional social services generated interest and motivated the adolescents to take part in DREAMS.

### Community-level factors facilitating adolescents’ participation in DREAMS interventions

#### Involvement and support of community leadership

The community leaders played an important role in supporting the local CBO in delivering DREAMS interventions by encouraging community members including grandparents, through community meetings, to allow the young people to participate in DREAMS. Additionally, the community leaders reached an agreement with the local CBO to hire people from the community in order to increase employment opportunities for local people and to facilitate better linkages with the organisation on any matter regarding DREAMS interventions.

*The community leaders wanted local people to be hired so that it would be also easier to reach our organisation in case of problems in the implementation of DREAMS in the community*. *(Let’s Talk intervention facilitator*, *female*, *41yrs*)

The involvement and support of community leaders were critical enablers of the continued participation of adolescents in DREAMS interventions, and the leaders articulated their demands to the CBO to maintain social cohesion and community economic development through the employment of local people.

### Individual-level factors hindering adolescents’ participation in DREAMS interventions

#### Diverse learning styles

Diverse learning styles among adolescents hindered learning in the behavioural interventions for HIV prevention. Two adolescents expressed challenges they faced due to the learning methods which were used to deliver DREAMS interventions. One adolescent narrated his challenge of participating in Stepping Stones intervention:

*There is something I was not comfortable with and it scared me*. *I am known as a person who doesn’t like talking*. *However*, *there* [group sessions for Stepping Stones], *I had to talk in order to relate with other people*. *I was not free to talk about things like male body parts in a group setting*. *… I also didn’t like doing role plays about violence against women and girls … I don’t like violence and it felt like I was being violent*. *(Thabani*, *boy*, *15yrs*)

It was clear that Thabani struggled with role-play scenarios depicting gender-based violence (GBV) and was not comfortable with sharing his personal experiences about sexual organs within a group setting. However, during the interview, he shared that he never experienced any form of GBV in real life.

On the other side, the other adolescent who participated in Let’s Talk and Vhutshilo interventions shared her frustrations with participating in written tasks as she preferred oral activities:

*I enjoyed DREAMS when there were no written tasks*. *I preferred group discussions … I easily get tired of writing*. *Sometimes*, *I didn’t feel keen to attend group sessions because I wanted to avoid writing activities*. *(Thandi*, *girl*, *13yrs*)

It appeared that the written tasks in DREAMS hindered participation and thus affected her willingness to actively engage in a meaningful way.

#### Internalised stigma of being labelled as ‘badly-behaved’

Internalised stigma of being labelled as ‘badly-behaved’ hindered adolescents’ participation in DREAMS. It was mentioned that being a sexually active adolescent was prohibited and thus labelled as ‘badly-behaved’. Mocking of pregnant girls, both at school and in the community, generally hindered their participation in services as they opted to isolate themselves.

It was also mentioned that some pregnant adolescents dropped out of school due to stigma and teenage mothers did not participate in DREAMS interventions to avoid being labelled as ‘badly-behaved’. This account exemplifies the internal challenges and battles adolescents faced once they became pregnant and labelled as ‘badly-behaved’. The DREAMS intervention facilitator explained that there was a misconception that DREAMS was targeting the ‘well-behaved’ young people. However, the facilitator mentioned they consistently corrected the misconception by emphasising that:

*We enrolled and accepted both adolescents who had babies and those who did not have babies*. *We even told those who were teenage mothers how to prevent unplanned pregnancies*. *(Let’s Talk intervention facilitator*, *female*, *41yrs*)

Tension arose when the DREAMS intervention engaged those who were labelled as ‘badly behaved’ and, thus, facilitated hesitancy and internalised stigma from others to participate in DREAMS.

#### Obtaining fragmented Sexual and Reproductive Health (SRH) information

Some adolescents mentioned that they received fragmented SRH information between their churches and DREAMS. They reported that they were taught about contraceptives and different types of contraceptives to prevent unplanned pregnancies and HIV in DREAMS. In contrast, their churches prohibited premarital sex and thus the use of contraceptives by youth. One adolescent narrated teaching from his church:

*They say men should not have sex before marriage*. *Women should also not*, *and they should ensure they do not have sex before marriage*. *In church*, *they make it clear that if you fail* [to abstain], *come to church for prayers … but in DREAMS*, *they told us about condoms*. *(Thabani*, *boy*, *15yrs*)

Some adolescents also indicated that their churches were silent about HIV and instead they were taught about abstinence:

*In church*, *we are taught about abstinence … nothing is said about HIV and contraceptives … yet we learnt about HIV from DREAMS but not from our church*. *(Thandi*, *girl*, *13yrs*)

*No*, *in church they don’t teach us about HIV*. *(Neli*, *girl*, *14yrs*)

Further, the church messages were also advocated at home by the older carers. It was clear that adolescents were conflicted with messages they received from their church, home, and DREAMS. Messages that reinforced what the adolescents knew, based on church and home teachings were easier to absorb than those that conflicted with normative thinking related to abstinence and virginity.

### Interpersonal level factors hindering adolescents’ participation in DREAMS interventions

#### Negative peer pressure

Negative peer pressure emerged as a barrier to participation in DREAMS for some adolescents. This was illustrated by one adolescent as she discussed that she experienced negative peer pressure as her friends demotivated her from participation:

*My friends decided to withdraw from participating in DREAMS*. *They said that they felt bored during group sessions*. *Then*, *they expected me to give up participating in the programme*. *I simply ignored them*. *Sometimes*, *they stopped me on the way to attend classes*. *(Zama*, *girl*, *15yrs*)

Despite the adolescents stating they enjoyed attending DREAMS with their friends, the experience was different for this adolescent whose friends withdrew their participation and thus expected her to pull out from DREAMS.

#### Refusal by grandparent caregivers or parents

DREAMS intervention facilitators reported that some adolescents in the community were not allowed by their grandparent caregivers or parents to participate in DREAMS. Two reasons were mentioned for refusals. Firstly, some adolescents were not allowed to participate in DREAMS as a punishment for not carrying out household chores. Some caregivers made restrictions that adolescents were only allowed to attend DREAMS sessions once they had completed the household chores:

*Sometimes household chores commitments prevented them from attending group sessions*. *Apparently*, *some young people came to attend sessions whilst they had not properly completed their assigned chores at home*. *We would then enforce it to the young people ‘you should do 1*, *2*, *and 3* [meaning household chores] *because your parents expect you to do that before you come for group sessions’*. *(Stepping Stones intervention facilitator*, *male*, *30yrs*)

Secondly, another DREAMS intervention facilitator mentioned that some adolescents left their homes as if they were going to attend DREAMS, but instead went to hang out elsewhere. When caregivers learnt about these lies, they immediately refused to allow the adolescents to continue participating in order to punish them for lying.

#### Caregivers’ lack of information

A lack of information by older caregivers about DREAMS hindered young people’s participation in the interventions. This was illustrated by two older caregivers:

*One day*, *he told me that he is attending classes* [DREAMS], *but I didn’t know what was taught to him*. *Whenever he came back from school*, *he would say ‘they are calling me*, *I’m going there*. *There is something we are learning’*. *Initially*, *he would not say what it was that they were learning*. *Eventually*, *he said they were learning about HIV after I had asked him several times*. *(MaNdawo*, *older carer*, *76yrs*)

*They did not give me an explanation*. *… One day I asked*, *‘what are they teaching you because you do not explain anything to me’*? *I was worried that I didn’t have detailed information about conversations Sane was part of*. *(MaZulu*, *older carer*, *64yrs*)

It appeared that while the grandparents provided permission to the adolescents to participate in DREAMS, the intervention facilitators may not have provided sufficient information for the grandparents to comprehend the content of information to be received by adolescents during the HIV prevention interventions. Therefore, the grandparents needed detailed explanations from their grandchildren who seemed not keen to share openly with their grandparents, unless the grandparents were persistent in finding out from the adolescents. The lack of information limited the support that the older carers provided to the adolescents who were participating in DREAMS and compromised the care relationships.

### Organisational level factors hindering adolescents’ participation in DREAMS interventions

#### Limited financial resources

We found that limited financial resources to reach the many adolescents who needed HIV prevention interventions hindered adolescents’ participation in DREAMS. This was reflected in the explanation by the DREAMS intervention facilitator:

*Enrolling 20 adolescents in this ward does not mean that only 20 adolescents are living in this ward*. *They are many who could not receive the interventions because each group was limited to 20 adolescents*. *(Stepping Stones intervention facilitator*, *male*, *30yrs*)

Further, limited funding constrained the organisation from hiring sufficient programme facilitators to facilitate planned programme sessions in the various sites. It was indicated the intervention facilitators were overwhelmed by the demand to facilitate the number of groups in the different sites. The facilitators felt that the workload and pressure to meet targets, sometimes made it difficult for them to be punctual for the planned group sessions with the adolescents. In turn, disruption of the group sessions frustrated the adolescents, especially when there was no communication about delays or cancellations of the programme sessions.

*I didn’t like waiting for the facilitators because*, *sometimes*, *they would arrive late and not send any communication*. *I also didn’t like when the sessions were cancelled*, *but I appreciate that they would apologise for being late to start the sessions or for making late cancellations*. *(Sane*, *girl*, *13yrs*)

Lastly, at the beginning of the interventions, some adolescents were provided with transport fare as an incentive to participate in DREAMS interventions. Many adolescents were motivated to participate as they were informed during recruitment about the incentive money. However, the organisation discontinued the transport fare due to the limited financial resources and upon realising that adolescents did not have to incur transport costs for participation in DREAMS as community-based sites were used. The facilitator explained:

*In the beginning*, *there was a transport allowance*. *Later*, *it was realised that we do group sessions in the community where they live*. *Therefore*, *there was no need for a transport allowance*. *So*, *the allowance was never paid to some adolescents*. *… There was noise about this in the community … since young people were recruited on the premise that they will receive money which some did not get*. *(Let’s Talk intervention facilitator*, *female*, *41yrs*)

Some adolescents who did not receive the transport fare were not happy about it. They expressed their feelings:

*Ay*, *there*, *they lied to us*, *there*! *Oh*, *my goodness*, *they lied to us*! *They said we must join the classes; they will give us money*. *Each day of attending will accumulate R10*. *We never received our money until now*! *(Zama*, *girl*, *15yrs*)

*Oh*, *there*! *They told us we would get money for attending classes*. *… They played us*. *You see*, *people are saying ‘they will not go to attend if the classes start again’ because they were told lies’*. *(Sane*, *girl*, *13yrs*)

It was clear that the adolescents were unhappy about not receiving the transport fare as promised and regarded it as dishonesty on the part of the organisation implementing the DREAMS programme.

#### Characteristics of DREAMS interventions

Some aspects of the DREAMS interventions were identified as hindering successful adolescent participation in the HIV prevention interventions. Six intervention characteristics were reported.

The first characteristic regarded DREAMS interventions that were school-based and required the participation of adolescents within selected schools. One adolescent was upset that not all schools in the community were selected for DREAMS. As a result, she could not spend time with friends whose schools were not selected in the intervention and was forced to walk home from school alone:

*After school*, *I had to remain to attend DREAMS sessions*, *while other girls from* [neighbouring school] *walked back home*. *We walked together after school*. *So*, *I had to walk alone back home because their school was not part of DREAMS*. *Some are my friends and I lost time to be with them*. *(Thandi*, *girl*, *13yrs*)

The second characteristic that was a major barrier to adolescent participation was the exclusion of adolescent boys in the Let’s Talk intervention. The intervention was only for adolescent girls and their parents/caregivers. One facilitator stated that the exclusion of boys in the intervention influenced HIV risk for adolescent girls:

*The programme was not supposed to include girls and their caregivers only*, *but it should have been the caregivers and their children*, *both boys and girls… boys also needed the intervention that the girls received*. *Girls were taught about the programme*. *They were taught about how they can protect themselves from HIV*. *They were taught about teenage pregnancy*. *The problem is that a girl does not impregnate another girl*. *A girl does not acquire HIV from another girl*, *unless through sores*. *A girl gets pregnant due to having sex with a boy*. *A girl acquires HIV from having sex with a boy*. *A girl does not have powers over a boy*, *but the boy has powers over the girl*. *… If a girl refuses sex*, *the boy would force a girl until she has sex with him*. *… Girls were taught how to behave*, *but boys were not*. *In the programme*, *we talked with girls and their caregivers whereas the boys were not there*. *Teaching boys would protect girls*. *If both boys and girls are taught*, *they would know how to build love relationships without sex*. *By so doing*, *teenage pregnancy and the spread of HIV can be prevented*. *(Let’s Talk intervention facilitator*, *female*, *41yrs*)

The third barrier was the exclusion of adolescents with disabilities in the HIV prevention interventions. One facilitator mentioned that adolescents with disabilities were overlooked for participation in DREAMS. None of the adolescents with disabilities were reached by the CBO delivering DREAMS interventions to receive the HIV interventions. Reasons cited for the exclusion were that these adolescents lived in boarding facilities and returned home during school holidays when it was also a recess for DREAMS intervention activities:

*I did not have* [adolescents with disabilities] *in my groups … most of them live in boarding schools for learners with disabilities*. *… It was not noticeable that they are not receiving the interventions because we delivered programmes during school days*, *while they come back home over school holidays*. *We were not active during the school holidays*. *(Let’s Talk intervention facilitator*, *female*, *41yrs*)

*Honestly*, *we did not pay attention to recruiting young people with disabilities*. *Of all the groups I facilitated*, *I did not have any young people with a disability*. *We do have them in the community but we did not make any effort to include them*. *Even when reporting*, *we were not asked about reporting recipients with disabilities*. *(Stepping Stones intervention facilitator*, *male*, *30yrs*)

The issue of exclusion of adolescents with disabilities from the DREAMS intervention was also raised by some older carers.

*I have a grandson who has mental health disorders as result he dropped out of school*. *My problem is that he is just sitting here at home without anything to do*. *I wanted him to join the classes* [DREAMS] *but they did not take him*. *(MaNgubo*, *older carer*, *80yrs*)

*My younger granddaughter has a hearing disability and is no longer attending school*. *She is also not earning a child support grant because I don’t have money to take her to the doctor for assessment in order to apply for the support grant … she did not attend the classes* [DREAMS]. *How was she supposed to go there when she is deaf*? *I don’t think they were going to allow her*. *I don’t think they took children with disabilities*. *(MaJali*, *older carer*, *56yrs*)

The fourth barrier associated with characteristics of DREAMS interventions was the exclusion of older carers in the Let’s Talk intervention. Some older caregivers were concerned that they were excluded from participating in the intervention with their adolescent grandchildren due to age and physical abilities.

*I did not attend the classes with my granddaughter*. *She* [programme facilitator] *preferred my daughter* [Neli’s aunt] *to come with Neli*. *I even asked her* [programme facilitator], *‘how does it happen that I don’t go with my granddaughter yet I’m the one caring for her*?*’ She simply said ‘I thought you would not afford to attend every session because you are also looking after the house and the young babies’*. *Then*, *I did not attend the classes*. *(MaZulu*, *older carer*, *64yrs*)

*I could not attend the classes because she* [programme facilitator] *said ‘I’m too old’*. *They took my daughter* [Zama’s aunt] *instead of me to go with Zama*. *(MaNgubo*, *older carer*, *80yrs)*.

The intervention facilitator explained that they excluded older caregivers to save them from the strain of walking to the venues:

*We excused older caregivers*, *especially those who are too old and unable to walk … we decided that their adolescent grandchildren must attend the sessions with someone who is still physically active and able to walk to the venues*. *We also thought it was going to be too hectic for the older people to abandon their housework and come to attend the sessions*. *(Let’s Talk intervention facilitator*, *female*, *41yrs*)

Fifth, the timing of programme sessions was not convenient for some adolescents to attend the DREAMS interventions. Participants reported that DREAMS sessions that were scheduled for late afternoons conflicted with adolescents’ housework commitments.

*Sometimes*, *the young people could not come for sessions that were held in the late afternoon*. *This is the time most of them are busy with household work such as cooking*. *(Stepping Stones intervention facilitator*, *male*, *30yrs*)

*I missed a few sessions because they were late afternoon*. *… I was unable to attend the late afternoon sessions because I need to cook before it gets dark*. *(Mpume*, *girl*, *19yrs*)

Lastly, recruitment strategies employed by the CBO delivering DREAMS interventions hindered the participation of some adolescents in the HIV interventions. The intervention facilitator explained that the Let’s Talk intervention was community-based and used a door-to-door recruitment strategy whereby caregivers with adolescents aged 13–19 were recruited first. Often, these adolescents were in school while their caregivers were recruited. On the other hand, the Vhutshilo intervention recruited adolescents aged 10–14 directly in schools. It was explained that the Let’s Talk intervention was unable to meet its target as some adolescents were already participating in the Vhutshilo intervention.

### Community-level factors hindering adolescents’ participation in DREAMS interventions

#### Youth empowerment a potential threat to intergenerational power dynamics

Participants stated that DREAMS interventions were also empowering youth and, thus, viewed as a potential threat to intergenerational power dynamics. At first, it was mentioned that some community members perceived the interventions as ‘*a thing for women*’ and others labelled the interventions as a campaign to teach young people to control adults:

*It was sometimes difficult to recruit young people in the community to participate in DREAMS*. *We met resistance from some people*, *especially men*. *Some men in the community called DREAMS a thing for women and didn’t want to listen to us*. *Others said*, *‘Your women thing annoys us because you teach children to control us’*. *They felt DREAMS came to teach young people to be disrespectful towards adults*. *(Let’s Talk intervention facilitator*, *female*, *41yrs*)

The perception that DREAMS was disrupting generational power dynamics between adults and young people hindered adolescents’ participation in DREAMS.

#### Norms of sexual communication

Norms of sexual communication in the community were identified by the DREAMS intervention facilitators as affecting their ability to facilitate some sexual health topics with the adolescents as these topics clashed with community norms around sex and sexuality:

*Sex topic*! *The reason it was difficult (laughing) for me is my church position*. *I am a pastor’s wife*. *Many people create a wrong picture that since I am a Christian*, *I do not talk about that* [sex]. *It was difficult to utter sex-related words*. *When I said those words*, *all participants closed their mouths* [indication of surprise] *and said ‘even you can utter such a word*!*’ Sometimes I felt uncomfortable after talking about sex and regretted discussing the sex topic*. *However*, *I ignored the feelings because it was my job and I*, *as a facilitator*, *was required to talk about sex with young people and their caregivers*. *(Let’s Talk intervention facilitator*, *female*, *41yrs*)

*You see*, *the topic which was difficult for me to facilitate is the topic of menstruation*. *It’s the only one*. *I tried as I was expected to facilitate it*. *I was not familiar with facilitating it*. *… I never thought I would find myself facilitating this topic*. *The topic was difficult for me as a man to talk about menstruation in the midst of girls … it’s not common for a man to talk about menstruation in the presence of women because we*, *as men*, *don’t menstruate*. *People think it’s not our topic to discuss*. *(Stepping Stones intervention facilitator*, *male*, *30yrs*)

It was clear that norms of sexual communication challenged the intervention facilitators when discussing sex and sexual development with young people. However, the young people did not express any problems with the intervention facilitators discussing sex, except, as stated before, for one adolescent boy.

## Discussion

In this poor and rural community hard hit by HIV, DREAMS interventions strengthened the life skills of adolescents in older carer families, particularly interpersonal relationship skills, and coping with emotions. The impact was evident both within the complex family dynamics and through redefining and recognising healthy peer relationships. This was most effective when DREAMS messaging reinforced what adolescents and older caregivers believed to be existing norms around sex and sexuality and when it was delivered by trusted community members in a familiar space. DREAMS was less acceptable when it deviated from these norms, raised SRH information that conflicts with abstinence and virginity and when youth empowerment was perceived as a potential threat to intergenerational power dynamics. While it is reassuring that DREAMS was able to engage these complex families, there were failures, in relation to factors uniquely critical to these families, such as in engaging children and carers with disabilities and failure to engage those busy with chores, particularly in the context where adolescents may themselves be caregivers of their older carers.

Family, peers, and HIV programme facilitators were instrumental in supporting the participation in HIV intervention by creating an enabling environment for adolescents to participate. These aspects are at the interpersonal level of the SEM. This is consistent with other studies showing that support from significant others has a positive impact on adolescents’ uptake of health promotion interventions [44]. The support that adolescents received from their peers and older carers motivated the adolescents to participate in DREAMS interventions. However, refusal by caregivers and the lack of information given to caregivers negatively influenced adolescents’ participation in HIV interventions. The results show that relationships with caregivers played an important role in the participation of adolescents in HIV interventions. Disapproval and lack of support from significant others such as caregivers and peers may limit access to HIV interventions by adolescents. Families are well-positioned to reinforce motivation, decision-making, and adolescent protective behaviours [45]. HIV interventions need to consider the influence of caregivers and therefore promote communication between adolescents and their caregivers. In addition, it is imperative to involve and empower older carers with knowledge about SRH, HIV, and AIDS.

As stated before, domestic work seems to influence care relationships between adolescents and their older carers. In addition, domestic work responsibilities also influenced the participation of young people in DREAMS interventions. These findings mirror those of other researchers that domestic responsibilities were barriers for adolescents in accessing HIV interventions [46]. In this study, the older carers expected young people to prioritise domestic chores over participation in HIV interventions. Refusing to let them attend DREAMS sessions was used as a punishment for not performing domestic chores. While it may be important for young people to perform their domestic responsibilities, this may give the impression that HIV interventions are less important than domestic work. A study in Uganda reported that children from older carer families were often coming late to school as a result of domestic chores [1]. These findings show that domestic tasks are a burden for the adolescents in older carer families and this needs attention as it may compromise their health and well-being.

Moreover, in this study, improved care relationships and communication between adolescents and their older caregivers as a result of the exposure to DREAMS programme components generated interest in the interventions and motivated adolescents to participate in the HIV interventions. According to study participants, DREAMS interventions provided a specific and critically important experience of improved care relationships between adolescents and their older carers. As participants described these, they reflected on connections and bonds that are critical to adolescents’ well-being. The DREAMS Let’s Talk intervention mitigated communication barriers between adolescents and their older carers. Other studies in South Africa have similarly found that a caregiver/parenting programme improved interaction between adolescents and their primary caregivers and facilitated important conversations about sensitive topics [42, 47, 48]. It seems the DREAMS Let’s Talk intervention promoted sexual health communication between adolescents and their older carers and reduced adolescents’ behavioural risks. For example, one adolescent reported delaying engaging in sexual intercourse as a result of communication with her older carer that the programme facilitated. With a clear interest in participating in DREAMS and a stated benefit by some participants for promoting sex communication between adolescents and grandparents, these findings also suggest that DREAMS acted as a platform for young people to discuss sex with adults. Findings from another study in South Africa have described this as a positive outcome whereby the intervention created space for open and meaningful conversations about sex between parents and young people [49]. However, the study raises important issues about the lack of sustainable communication between adolescents and older carers after the completion of the DREAMS programme. Previous research from a family-based HIV prevention intervention in South Africa has also indicated a decline in parent-child relationship quality post-intervention [50], which is an important protective factor for adolescent risky sexual behaviours [17, 19]. Our findings highlight that increased efforts to enhance older carer-child communication about sex post-intervention are still needed. The question of how the communication between adolescents and older carers can be sustained after interventions have come to an end is a matter of great concern.

This study found that the implementing organisation was important in creating greater access to other services in addition to HIV prevention interventions. For example, the programme facilitators assisted and linked the older carer families with other social services to obtain birth certificates and to apply for child support grants. These findings showed that offering a combination of services which adolescents need, motivated their participation in HIV prevention interventions. Also, the findings of this study highlight the utilisation of lay facilitators to deliver HIV prevention interventions. The utilisation of lay facilitators is common in low-income settings [51–53]. While the utilisation of lay facilitators promotes employment opportunities for local community members, they may lack competent skills and self-efficacy to challenge community norms of sexual communication.

Moreover, conflicting sexual health messages and information between different structures confused the adolescents. The research reveals that adolescents obtained conflicting SRH information from their churches, homes, and DREAMS organisation. The contradictory information undermined the HIV prevention initiatives, promoted by a fragmented and uncoordinated approach between different social structures in fighting HIV. This study shows that confusion about conflicting SRH information may be influenced by aspects that occur at the interpersonal, community, and organisational levels of the SEM. Also, their beliefs about whether to use contraceptives and condoms or not were shaped by sources of information they obtained from their families, churches, and HIV implementers. This had important implications on the young people’s desire to participate in HIV interventions as attitudes and beliefs play an important role in the intention to carry out a behaviour [26, 28, 29]. These findings have implications for the design of HIV prevention interventions and highlight the lessons learned in this study that reinforcement of health messages between DREAMS, churches, and families facilitated positive behaviour change in adolescents [52]. Furthermore, the quality of collaboration between information providers and adolescents is also important to bring about positive change. We recommend that integration between community structures and HIV implementers is necessary for HIV interventions to be culturally responsive and acceptable.

Further, adolescents’ attitudes towards participation in the HIV interventions were influenced by how they were being labelled by peers, family, and in the community. Being labelled as ‘badly-behaved’ and internalisation of this label created significant barriers to adolescents’ participation in HIV interventions. It also led to internalised stigma. This is consistent with previous research in South Africa which has shown self-stigmatisation to be a barrier to engaging in HIV and health services by adolescents [54, 55]. Moreover, internalised stigma compromised adolescents’ self-esteem as they avoided engaging in HIV interventions with their peers. In this setting, a study investigating awareness and uptake of DREAMS interventions [56] also found that DREAMS was not effective at reaching AGYW who had ever had sex or ever been pregnant, which may reflect internalised stigma that created barriers for AGYW to participate in DREAMS. The authors suggest that interventions that focus on the individual level of SEM can be addressed by changing the current negative beliefs and attitudes that young people have about themselves as recipients of HIV prevention interventions. A study in Ibadan Nigeria reported that the best predictors for risky sexual behaviour are low self-esteem and low monitoring practices of parents or carers while lower levels of authoritative parenting were found to be associated with risky sexual behaviours [45]. These findings add to the body of qualitative research, which has suggested that internalized stigma contributes to anti-social behaviours and mental health problems [57, 58].

At an organisational level, the exclusion of boys in the DREAMS Let’s Talk intervention was a major barrier to the participation of adolescent boys in the programme. While excluding adolescent boys in the programme may affect family communication with their caregivers, it also placed adolescent girls at increased risk of HIV infection and unwanted pregnancies. Public health researchers have long noted that adolescent girls are disproportionately affected by HIV in SSA [8]. While more interventions are now in place to reduce HIV infections among adolescent girls, it seems adolescent boys are not prioritised. These findings corroborate with those reported in South Africa that exclusion of adolescent boys and young men in HIV prevention interventions is counter-productive, inequitable, and did not play a major role in reducing HIV incidence among AGYW [29]. Being left out of certain HIV prevention interventions may have detrimental effects on the sexual behaviours of adolescent boys. Consequently, adolescent boys may have the belief that they are not at risk, therefore, are less affected by HIV and AIDS. This may further reinforce gender stereotypes that women are responsible for SRH. Earlier work has pointed out the feminization of SRH services [59] and that men view women as responsible to seek reproductive services such as contraceptives while they have little negotiating power regarding their own reproductive health [60]. Moreover, adolescent boys may be ignorant of reproductive health matters and engage in risky sexual behaviours, making them susceptible to HIV infection and other STIs. Therefore, the findings of this study highlight the need for the inclusion of boys in HIV interventions. We support the importance of engaging men as clients in SRH interventions than single-focus interventions [61].

The study findings raise the important issue of exclusion of adolescents with disabilities in DREAMS interventions, education, and social welfare services. This is important because disability is often associated with stigma [62]. The DREAMS implementing organisation in this study had difficulty recruiting and enrolling adolescents with disabilities as recipients of HIV prevention interventions. Consistent with previous reports, adolescents with disabilities in South Africa still lack access to HIV interventions and SRH services [63–66]. The exclusion of adolescents with disabilities might also imply, among other things, that the DREAMS implementing organisation did not appreciate and acknowledge young people with disabilities as beings. A systematic review of studies in SSA reported that barriers to accessing healthcare services for young people with disabilities aged 15 years and above include attitudinal biases of health and social service providers and a lack of adaptation of health information to suit young people with disabilities [67]. Accessing HIV interventions and interacting with other young people may provide comfort to young people with disabilities. Being marginalised from participation in HIV prevention interventions may have detrimental effects on the sense of self-worth of adolescents with disabilities. Consequently, adolescents with disabilities may have the belief that they are asexual beings, therefore, unaffected by HIV and AIDS. Moreover, they may engage in risky sexual behaviours as a mechanism of self-esteem validation to overcome the belief of being asexual [63], making them prone to HIV infection and other STIs as well as pregnancy. A study in South Africa reported that 11.8% of adolescent girls aged 15–18 years with a reported disability were HIV positive compared with 3.3% of girls with no reported disabilities [68]. These results are worrisome considering that DREAMS interventions were implemented in the same district in which this study found the marginalisation of adolescents with disabilities. The findings have implications for future research to bring to light the socio-ecological factors that shape the participation in HIV interventions for adolescents in older carer families and in the general public.

Staying in the house all the time or not attending school may reinforce the social exclusion of these young people as they may have limited opportunities for education and for interactions with their non-disabled peers. The uMkhanyakude District, where the study was conducted, is the poorest rural district of the KwaZulu-Natal province. In 2016, the Section 27 study reported that there were 14 registered schools for children and adolescents with disabilities in the district, and of these, only one was a high school [69]. Furthermore, another study in the district reported that disabled children and adolescents aged 7–18 years showed higher proportions of not attending school (8.7%) compared with children and adolescents without reported disabilities (4.1%) [68]. The findings of this study add depth, in that some of these adolescents with disabilities are also in older carer families which raises a need to focus on these adolescents.

In addition, economic factors may also make adolescents with disabilities vulnerable to poverty. While the monthly disability social grant provided by the South African government makes young people living with disabilities financially better off, the findings of this study showed that older carers caring for young people with disabilities had challenges in accessing social grants. Findings presented here complement those reported in South Africa that only 5.6% of children and adolescents with a reported disability were recipients of disability or care dependency grants, while 21.9% were not receiving any support at all, despite having a reported disability [68].

## Strengths and limitations

The major strength of this study is its ability to identify the socio-ecological factors which are most critical to participation in HIV interventions among adolescents with grandparent caregivers by obtaining the perspectives of the adolescent-grandparent dyads and the programme facilitators. Our design takes advantage of the repeat interviews that enabled discussing and following up on issues that the study participants raised in the previous interviews. Interviewing dyads separately also enhanced understanding of care relationships and dynamics between adolescents and their older carers, from the perspective of each individual member of the pair (dyad).

One important limitation of this study is that the study sample comprised of one adolescent boy. This was because adolescents were recruited through one DREAMS implementing organisation and the researchers found only one adolescent boy who was a recipient of DREAMS interventions and being cared for by a grandparent caregiver. More studies are still needed to investigate contextual issues of adolescent boys with grandparent caregivers.

## Conclusions

In this study, we investigated the adolescents’ facilitators and barriers to participation in DREAMS interventions from the perspectives of adolescents, their grandparent caregivers and DREAMS intervention facilitators in rural South Africa. We found that care relationships could be an overarching barrier and facilitator across the four levels specified in the socio-ecological framework. The importance of this study highlights that continued efforts are needed to strengthen care relationships between adolescents and their grandparent caregivers, involve grandparent caregivers, and meet the needs of adolescents. This may lead to improvement in adolescents’ participation in HIV interventions and subsequently, mitigate adolescent risky behaviours. The findings presented here demonstrate areas of particular need across the SSA and provide a call to action for service providers, donors and policymakers, to think critically about the importance of providing services to adolescents with grandparent caregivers. Adapting HIV interventions to meet the needs of adolescents with complex family backgrounds is foundational to the success of achieving the UNAIDS 95-95-95 targets.
